# Tiritiria: Understanding Māori children as inherently and inherited-ly literate—Towards a conceptual position

**DOI:** 10.1007/s40841-023-00282-7

**Published:** 2023-04-15

**Authors:** Maia Hetaraka, Selena Meiklejohn-Whiu, Melinda Webber, Rebecca Jesson

**Affiliations:** 1 Te Kura Akoranga me Te Tauwhiro Tangata | Faculty of Education and Social Work, Waipapa Taumata Rau | University of Auckland, Auckland, New Zealand; 2 School of Curriculum and Pedagogy, Waipapa Taumata Rau | University of Auckland, Auckland, New Zealand; 3 Te Puna Wānanga, Waipapa Taumata Rau | University of Auckland, Auckland, New Zealand

**Keywords:** Literacies, Mana, Te Ao Māori

## Abstract

Many theories support the idea that children’s literacy learning develops as they learn to make meaning through interactions with others. These assertions are premised on the understanding that childhood literacy serves various social purposes and that these literacies are learned through participating in social contexts. In this position paper, we seek to reframe current, widely accepted understandings and definitions of literacy. We draw upon mātauranga Māori (Māori knowledge/wisdom) concepts to illustrate Māori philosophical views about the nature of knowledge production. These concepts clearly delineate the link between knowledge, literacies, and power, a link often actively overlooked by western framing of literacy. We use a Māori whakataukī (proverbial saying) to re-conceptualise current understandings of literacy, positing varied literacies and literacy practices. Within this conceptual framework Māori children are re-positioned as maurea – treasures of the highest order, born of and with mana, an integral part of generations of whakapapa (genealogy), and an essential element in an intricate web linking all things (human and non-human). This paper proposes that children are inherently and inherited-ly literate; they are born literate—inheritors of multiple and cumulative genealogies of multimodal communication and knowledge sharing.

## Introduction

This position paper offers a contextually and culturally grounded starting point for investigating literacies in Aotearoa, New Zealand. Our team are part of the international Northern Oral Language and Writing through Play (NOW Play) project consortium (https://now-play.org/). The NOW Play project originated in Canada and includes indigenous communities from Canada, Sweden, and Aotearoa New Zealand. The authors of this position paper form the Aotearoa branch of the project.

Dr Maia Hetaraka (Ngāti Wai, Ngāi Tahu, Ngā Puhi) is a lecturer in the Faculty of Education, Waipapa Taumata Rau, The University of Auckland, and is the Director at the Tai Tokerau campus. Maia has a professional background in primary and tertiary education and a research interest in mātauranga Māori and the complexities of its relationship with other knowledge bases, especially within the context of colonised society. Maia is particularly interested in the ways traditional indigenous knowledges can be used to solve contemporary challenges.

Dr Melinda Webber (Ngāpuhi, Ngāti Kahu, Ngāti Whakaue) is a Professor in the Faculty of Education and Social Work at the University of Auckland. She leads a number of school and community research projects focused on better understanding the effects of motivation and academic engagement, culturally sustaining teaching, localised curricula, and enduring school-family-community partnerships on Māori student flourishing at school.

Selena Meiklejohn-Whiu (Raukawa, Samoa, Pākehā) is a research fellow at the Faculty of Education, Waipapa Taumata Rau, The University of Auckland. Her professional experience includes teaching and facilitation across sectors. Her doctoral research examines the potentialities of digital interactions and authorship for Pacific students to explore their cultural and learner identities.

Dr Rebecca Jesson (Pākehā) is an Associate Professor in literacy education at the Faculty of Education and Social Work at the University of Auckland. Rebecca researches in Research Practice Partnerships with schools and their communities in Aotearoa New Zealand and the Pacific. Her focus is on designing, with teachers, teaching and learning innovations that will improve the literacy learning experiences of students.

We seek to challenge the dominant notion of literacy as it is widely understood in New Zealand English-medium schooling contexts, and to understand how literacies manifest in the early years of primary education for Māori children.

Underpinning all of the phases of the emerging conceptual framework outlined in this paper, as well as the multifaceted experiences of literacy is our focus on the meaning making carried in language. Thus, language is learned, it is the tool for learning, and a primary vehicle of knowledge. This is illustrated by a whakatauakī (phrase/saying attributed to a speaker) of Sir James Henare stating, “Ko te reo te mauri o te mana Māori. Language carries the life force of our mana as Māori.” Language and literacies are the carriers of the philosophical and epistemological ideals of a culture.

For many Māori and other indigenous peoples, this is the reason why learning the language of the coloniser has been so incredibly damaging—language and literacy practices have been the carrier of philosophical ideals that have positioned us as inferior in every way. The displacement of indigenous social and cultural structures has been successfully achieved globally by displacing the languages that have transported epistemologies through time and space. This forms the basis of our argument that in Aotearoa New Zealand, it is time to reframe/re-conceptualise the ways in which literacy is understood.

## Reclaiming indigenous knowledge to re-conceptualise literacies

Western schooling, with its unwavering reliance on its own norms and definitions, has indoctrinated generations of Māori and non-Māori to believe that Māori culture is an illiterate culture (Derby, 2022; Walker, 2016). This belief positions Māori in very specific, deficit ways in a society and education system that judges academic ability on the arbitrary, narrow, western literacy norms of reading and writing (Hetaraka, 2020). However, Stewart (2013) asserts that “…literacy of the highest order is an act of pattern-seeking with a complex and incomplete array of words and numerals…” (p. 184). This definition provides a platform from which to challenge claims that Māori had no form of written language in that whakairo (carving) and raranga (weaving) are the complex arrays by which Māori have recorded and preserved Māori epistemology and histories. These also represent indigenous conceptualisations of the nature of knowledge and the universe through time and space.

In education, knowledge production processes have been dominated by western philosophical beliefs and worldviews. Elabor-Idemudia (2011) argues that these beliefs have undermined indigenous philosophical thought. In an ancient tauparapara (prophetic incantation often with hidden/dual meaning) that speaks specifically about knowledge production and dissemination the words ‘ka tiritiria’ describe a Māori philosophical view about the nature of knowledge production. Tiritiria refers to the way in which knowledge is passed from the spiritual realm into the physical, “it falls as raindrops do, to splash upon the earth” (personal communication, Te Warihi Hetaraka, September 11, 2014). From this perspective, knowledge is produced externally on a spiritual plane; it is interpreted by tohunga (person with highly specialised skill sets, chosen expert) then critiqued, constructed, re-constructed, and internalised under tapu (sacred, set apart) on the physical plane.

This worldview perceives knowledge as being so vast and varied that having only one way of knowing is unfeasible, a notion often rejected by dominant western philosophy that perceives its way of (all) knowing as superior. According to the notion of *tiritiria,* no one person, or even one group, could ever grasp all knowledge or ways of knowing—just as it would be impossible to stand in the rain and catch all the raindrops. Tuhiwai-Smith (2012) concurs that cultural knowledge systems contain multiple ways of knowing and multiple traditions of knowledge.

There are many differences between western and indigenous ways of knowing. Meyer (2008) argues that indigenous ways of knowing “…expand[s] the idea of what knowledge is supposed to be and in truth is—vast, limitless, and completely subjective” (p. 218). In contrast, when knowledge is consistently framed in a singular, ‘objective’, western dominated way, subjectivity is framed as ‘unknowing’, often used to discredit multiplicity.

In traditional Māori society, bodies of knowledge and belief systems were vast and spread throughout iwi under the protection of tohunga (expert), rangatira (esteemed leader, chief), and the general public—depending on the type of information (Hetaraka, 2020). Recognising the sacred nature of many forms of knowledge meant it was treated with respect and protected in ways that differ from western and contemporary methods. Its protection was to ensure its longevity for the benefit of the people. Māori views of knowledge and knowledge systems have been met with forms of gatekeeping by the western academy that have excluded, othered, and cherry-picked Māori knowledge according to the norms, values, and western perspectives of usefulness. It is with the view of knowledge from the perspective presented by the philosophy of tiritiria—that knowledge and knowledge systems are varied, context dependant and linked to practices within environments, that we seek to reframe literacies.

## Reframing literacies and literacy practices

Literacy can be understood as a vehicle or tool, encompassing the many ways that societies capture and share knowledge, through languages, texts, and communication. As outlined above, the knowledge that is shared, and the ways that knowledge is shared are social, and cultural. Thus, like many scholars before us, we argue that conceptions of literacy can never be neutral but are instead coloured by dominant cultural norms (Freire & Macedo, 2005; Gee, 2014; Luke, 2012). In Aotearoa, western schooling systems and curriculum prioritise dominant western culture and promote a singular view of literacy as an individual capability, developed in a linear way, evidenced through a narrow set of recognisable expressions (Milne, 2013; Pihama, 2019; Stewart, 2020). Therefore, Māori knowledge transfer processes (literacies) have been disregarded, misunderstood, and made largely invisible within the western education system.

Western education systems, and the literacies taught in them, have been used as a means of power to control society and environments (Jenkins, 1993; Pihama, 2019). Therefore, the power relationships that play out in societies are established and entrenched through literacy and literate practices. For example, literacy learning and theories are positioned within institutions such as schools, according to decisions that have been made about whose knowledge and communication tools are of value. Sutton-Smith (1951), a non-Māori author who studied play and games in Aotearoa communities, observed that in “the new cultural environment provided by the meeting of these two cultures [Māori & Pākehā], there has been a tendency for the unique pastimes of the submerged culture to be cancelled out…” (p. 107). Some of the inequities caused by submersions and power relationships can be addressed by developing deeper understandings of the ways thought, language, culture, and experiences influence literacy learning (Battiste, 2005; Gee, 2017). It is also essential to examine the spaces in which literacy is taught and learned.

As scholars have argued for many years, in Aotearoa New Zealand contexts, formal learning spaces are not neutral but reflect the norms and realities of the dominant culture (Milne, 2013; Pihama, 2019). Through ongoing marginalisation of te ao Māori, of mātauranga Māori and literacies, these misrepresentations have had an impact on children in the New Zealand school system (Pihama, 2019; Stewart, 2020). To further develop the ideas from this project the research team analysed three discrete data sets from Te Tai Tokerau (Northland, New Zealand). The data included kaumātua (elders), whānau (extended family), student and teacher voices. Perspectives about literacies and literacy practices emerged, revealing te ao Māori viewpoints that appeared to deviate from the more common western framing of literacies. These data and the analysis of literature forms the basis of the argument presented in this article—that indigenous perspectives provide robust ways to question, challenge and re-conceptualise status quo notions of what counts as literacy and valid literacy practices. We also posit that giving breath and energy to te ao Māori conceptualisations of literacies must happen in Aotearoa New Zealand schooling contexts to transform inequitable teaching and learning opportunities, and practices that continue to impede outcomes for many.

## An Emerging Theoretical Frame

The five components of the Mana Model (Webber & Macfarlane, 2020) were used during the initial analysis of the Tai Tokerau data. All five components—Mana Whānau (relationship and connection with others), Mana Motuhake (positive self-concept and a sense of embedded achievement), Mana Tū (tenacity and self-esteem), Mana Ūkaipō (belonging and connectedness to place), and Mana Tangatarua (diverse knowledge and skills) (Webber & Macfarlane, 2020, p. 26) were used to identify statements of success in the data. These sections of data were then analysed to understand how literacies emerge for Māori children. Following that process, we drew on traditional knowledge transfer processes (literacies) to explain the messages conveyed in the data from a Māori worldview. We used whakataukī (proverbial sayings) to shape our re-presentations of the data as core literacy principles.

Whakataukī (literally, ‘a saying to ground you’) are often commonly known Māori phrases relevant to a wide range of situations across time and space. It could also be argued, as it is by one kaumātua (elder; person of status within whānau) whose voice has been influential in this study, that whakataukī are small treasures left by tūpuna (ancestors) to be picked up by the people who need them, at the time they need them, for guidance, encouragement, and comfort (Hetaraka, 2020).

Whakataukī have the power to focus and/or redirect, they can also support the translation of complex philosophical understandings across cultures. Whakataukī serve a similar purpose in this position paper—they have helped the authors to ground their processes of understanding childhood literacies in Māori ways of being, doing and thinking. The whakataukī that revealed itself as relevant to this work is one that relates to progressions of human consciousness. We have used this version to support our development of conceptualising literacy teaching and learning from a te ao Māori perspective:

“Mā te rongo, ka mōhio; mā te mōhio, ka mārama; mā te mārama, ka mātau; mā te mātau, ka ora”

Through resonance comes awareness; through awareness comes understanding; through understanding comes knowledge; through knowledge we flourish.

Like many whakataukī, this one has its origins in ancient takutaku (pre-colonial incantation/prayer). Longer versions of this whakataukī describe each phase of the inception of the universe, which is argued by some indigenous knowledge holders to be reflected in the development of human intelligence—both individual and collective intelligence. The segment of the whakataukī presented here is done with the intention of supporting and illustrating one te ao Māori philosophy of the process of knowledge development. From this perspective knowledge is intertwined so closely with what we argue are literacies and literacy practices that to become literate in this way is to become fully conscious (another whakataukī presents full human consciousness as te whei ao, te ao marama, the world of light).

## Mā te Rongo: Resonance

Mā te Rongo is a phase of consciousness associated with a child’s need to experience and learn with all senses, perception, and awareness. This includes learning through rongo-ā-manawa (intuition). In te reo Māori, rongo is a word that encapsulates sound, smell, touch and vibration, so Ma te Rongo is a phase of learning that can happen without words, without specific instruction, it is intuitive. At the core of Ma te Rongo is the belief that a child has unlimited potential, which is ignited through the use of human senses.

The Māori philosophical belief that all children possess unlimited potential influences both our position on knowledge and literacies, and our consideration of Māori children. In this paper children are positioned as maurea (unique treasures), a term that in pre-colonial times was only attributed to humans (as opposed to the term ‘taonga’, which was used to describe non-human treasures). Here we conceptualise children as maurea, as a way of embedding the traditional Māori perspective that children are the most treasured source of our adoration, and the recipients of our utmost affections. In pre-colonial times, considering children to be maurea was to prioritise them within iwi. The future health and wealth of the people relied on the health, strength, knowledge, and mana of children. These concepts connect Ma te Rongo with Webber and Macfarlane’s (2020) concept of Mana Whānau, which as previously stated was used as an analytical tool in this project. In the phase of Rongo, children observe, listen, communicate, connect, and engage. Many believe that this learning phase begins in the womb at the point of conception (Hemara, 2000; Penehira, 2011), which causes the first reverberation needed to ignite the senses. The role of significant others around children (i.e. whānau) is also rongo – to observe, listen, communicate and engage, to ensure that, as they reveal their uniqueness, relevant and appropriate learning experiences can be presented to them.

According to Hemara (2000) the process of preparing a child for their education begins whilst the child is still in the womb. High levels of observation occur from pregnancy, and well into childhood, to ensure each child is provided with learning experiences that work with their individual strengths. This was carried out by māreikura (women of high rank) with the utmost care and investment. The role of māreikura has also informed our interpretation of children as maurea, the objects of our care and extreme attention. In thinking of children as maurea, there is an inherent understanding that they are innately connected to the world around them – spiritually, mentally, emotionally, and physically. This is a perspective of children not always applied or apparent in schools or in western literacy practices. In fact, it is common practice to compartmentalise literacy learning into discrete aspects, for example reading, writing, spelling. Teachers and students are both then faced with the challenging task of transferring knowledge and skills from one compartment to the next.

It is common practice in western schooling to disconnect students from the world around them, and from multiple ways of knowing. Brayboy and Maughan (2009) assert that all indigenous peoples “have their own ways of knowing, being, valuing and living in this world” (p. 391). Mā te rongo gives space back to children to feel and grow into ways of learning and knowing that suit their strengths. Considering an indigenous worldview of literacy development that demands space and time to engage all the human senses, and to practice focussed observation would be to expand currently widely accepted, western dominated versions of knowledge and literacies. An expanded view may empower children to explore more deeply, to bring their strengths, beliefs, and intuitive knowledge to their play spaces and, therefore, their learning. Mā te rongo activates multiple ways of knowing, being and living, which continues to be a systemic challenge in New Zealand education.

Aotearoa New Zealand’s education history shows that Māori ways of raising and educating children have been actively and purposefully devalued and positioned negatively within the curriculum (Hetaraka, 2022). What ‘counts’ as literacy is conveyed through curriculum, practices, assessments, and policies, where the “cultural practices of the dominant group are taken as the norm” (Gutiérrez & Rogoff, 2003, p. 19). These norms are relatively narrow and exclude indigenous literacy practices. The authors understand that correcting the injustices created by this approach requires multifaceted responses. One response presented in this paper, proposes an expanded view of literacy, by considering multiple ‘literacies’ with the intention of disrupting the ongoing messages of power and subordination transmitted by culturally dominant, narrow practices.

In western contexts, when children enter school, their own childhood, social and cultural literacies encounter the power-laden processes of western education. Bourdieu, (2004) refers to the valuing of the context as ‘cultural capital’, while González et al., (2005) speak about building on the child’s existing ‘funds of knowledge’. Children are quickly exposed to the power of texts, to shape what is received as ‘truth’ in official worlds (Dyson, 2003). When the capital the child brings with them matches that capital of the school, the child’s experiences are reinforced through texts, and their funds of knowledge are considered capital. However, when a mismatch occurs and funds of knowledge are not identified, acknowledge or extended, the cultural riches students bring to schools are not considered of value and then students experience what it is to be power-less in an incredibly powerful and tenacious system.

According to our developing framework, Mā te Rongo frames literacies not as tools by which to shape, or rather reinforce, official ‘truths’, but as tools for enriching children who already possess the internal resources for literacy learning. From a Māori perspective, Mā te Rongo encompasses all senses, including intuition. It allows knowledge to be “imparted in authentic and meaningful ways” (Glasgow & Rameka, 2017, p. 87). In traditional Māori society, knowledge was shared through playing a role, or role-playing daily chores (Glasgow & Rameka, 2017; Ritchie, 2012). It included children learning by doing and learning through experiences, often from everyday life. We propose that the role of adults in literacy teaching and learning at the Mā te Rongo phase is consistent with Genishi and Dyson’s (2009) identification of teachers as observers “who weave observation into interactions” (p. 118).

Observation according to Mā te Rongo is not using a set of criteria to judge student performance, nor to look for the sake of looking. It is to invest deeply to develop a strong sense of the child, it is to use all of one’s resources to intuit what the child needs, not to fill gaps, but to draw from and build on inherent strengths. Mā te Rongo encourages adults to trust their own capacity as an educator to develop and grow, through self-observation and the observation of children. Mā te Rongo ensures space for valuing the child for who and what they bring with them to learning. Mā te Rongo essentially activates energies that enable people to experience literacies and learning in multiple forms, it is the reverberation that will trigger the foundational pathways for future learning.

## Mā te Mōhio: Awareness

Mā te Mōhio refers to children becoming cognisant, aware, and agentic. Mā te Mōhio is to gift and be gifted opportunities to interact and act on the world. Mā te Mōhio offers possibilities, potential and foundations from which our children can be nurtured as they grow, in connection to all things around them as well as to themselves.

From a Māori perspective, knowledge and spirituality are inherently intertwined. Many Māori continue to perceive the physical world as immersed in and integral with the spiritual realm (Pere, 1982). Incorporating indigenous knowledges around spirituality is an example of seeking to repair the physical, material, emotional and spiritual damage caused to marginalised groups through practices of colonisation (Dei, 2011). An understanding of connectedness, and the implications of the damaged cause by forced disconnection, is important for Māori and non-Māori, as a necessary condition for us to act purposefully on, in and with the world.

Mā te Mōhio emphasises deep connection to people and place as an important part of teaching and learning. It also highlights the importance of local knowledge and learning from and within local contexts, places, people, and materials. Consistent with Webber and Macfarlane’s (2020) concept of Mana Ūkaipō, Mā te Mōhio suggests that these connections to places and spaces stir up, awaken, and connect to knowledge that maurea inherently and sub-consciously possess.

Whilst Māori conceptualisations of relationality are arguably somewhat broader and more interconnected (to time, space, place, people, universe) in comparison to western frames, they are congruent to socio-cultural theories of literacy learning. Formal western literacy education has often been problematic for indigenous and marginalised groups. Indigenous literacy practices, and therefore knowledge bases, have been diminished through western definitions of literacy. Consequently, Māori learners have found it difficult to connect with the learning materials and pedagogies, and many Māori whānau have been at a loss to know how to support their children’s learning at home. Mā te Mōhio encourages deliberate and purposeful use of contextual knowledge to support understanding of the ways in which diverse knowledges and literacies have evolved and continue to evolve through political, societal, and technological changes. The Mā te Mōhio phase of (re)conceptualising literacies encourage connections and conceptualisation to simultaneously broaden and deepen the learning that can be achieved through engaging in diverse knowledge sharing practices. It promotes an awakening in both broad and deep ways to help expand and reframe the current taken-for-granted understandings of literacies.

Māori literacies, knowledge production and sharing include a wide range of oral and visual practices, the use of narrative, ontology, and cosmology. At the intensive level of tohunga learning there is also a type of literacy that requires extreme degrees of concentration. These various literacy practices rely on a dynamic view of time and space, are inter-generational, linked to place (in a way that ripples forever outward, rather than being limited by place) and guided by cultural processes. In this paper, we propose that considering literacies, as tools for knowledge dissemination, from a Māori perspective has the potential to support a richer learning experience for Māori children.

With this proposed conceptualisation of literacy learning the Mā te Mōhio phase deepens learning through relationships to land, inter and intrapersonal relationships, and our relationships and responsibilities to environments and non-human relations. This re-centres te ao Māori ways of thinking and knowing. Drawing on mātauranga Māori literacies and practices children become grounded in a strong sense of who they are, which will inform decisions they make about where they may choose to go in their future. Rongo sparks the vibrations that set strong foundations for literacy learning, Mōhio awakens a desire to connect to people, places, and knowledges to link the pathways for learning with the ability to know.

## Mā te Mārama: Understanding

Mā te Mārama is to understand, comprehend and meta-cognate. Mā te Mārama includes new understandings, ideas, stimuli, and motivation to understand the workings of given stimuli. Mā te Mārama is the ability to process existing knowledge and to think abstractly in order to recognise connections and synergies.

Expanding understandings and discussions around literacies according to a Māori perspective, and with specific reference to Mā te Mārama includes broadening how we understand knowledge production and dissemination. As outlined previously, *tiritiria* reveals the process by which mātauranga Māori is disseminated, where no one person or group can possess all understandings and generate all new knowledge, but where every individual is born with their unique inherently and inherited potential to learn and generate knowledge (Mana Tū). From a Māori worldview the process of being in and impacting on the world is mutual, complementary, interdependent, and fluid. Literacy is therefore multifaceted and develops in ways that reflect the child’s connectedness, sense of belonging and membership to their whānau (extended family), hāpori (community), hapū, and iwi. Children are born whole—they inherit the mana and prestige of their ancestors and are destined to positively transform the world around them (Eruera & Ruwhiu, 2016). They are manifestations of the past, present, and future, simultaneously uri (descendent), tangata (person) and tupuna (ancestor). Literacy then, as understood from a Māori worldview, is always present and always developing, even before birth. Literacy (in its fullness) develops as children have opportunities to manifest and express mana.

Engagement in literacies and learning to ‘read’ a variety of textual forms comes through participation in many different cultural experiences, through engagement in meaningful interactions with oral, written, and visual texts. These interactions are contextual and relational, and many children learn by listening, watching, and eventually imitating other people and/or roles they observe (Glasgow & Rameka, 2016). They may also learn by trying things out for themselves, by testing their emerging ideas, by problem solving and by taking risks.

## Mā te Mātau: Knowing

Mā te Mātau is to know, to grasp, catch, and refine knowledge in order to harness it as practically useful. In Mā te Mātau children are secure in and confident of their place in the world. There is awareness of their connections and relationships to places, people and things around them. Mātau is the ability to transfer understandings and apply them to diverse (practical) situations, and produce new knowledge. To mātau is to have both the desire and the appropriate literacy tools and practices to experiment on and with current knowledge, to create new knowledge.

Traditionally, for children to contribute to and thrive in their communities, it was essential for them to develop strong socio-ecological literacy. These literacies were developed from bodies of knowledge collected over long periods of time, knowledge that was added to and changed as new generations made new observations, as people migrated, and needs and resources changed. This education was experiential, place-based but also dependant on the cosmological knowledge protected by tohunga—an illustration of the connection between the physical and spiritual. A key argument presented by the re-conceptualisation of literacies aligns with Sir Patu Hohepa’s belief that “[l]iteracy is more than just looking at words in a book; it is also about reading the world around you.” (Hetaraka, 2008, p. 29).

This assertion from Sir Patu Hohepa allows a glimpse of what literacy means from a te ao Māori perspective. The practices and processes of literacy teaching and learning are dynamic and contextual, they are as much about non-verbal communication as verbal. To become literate from this perspective is to have an awareness of identity (Mana Motuhake) and potential impact in relation to the world around us (Mana Tangatarua). The process of becoming literate from this perspective is also arguably far more intensive than becoming literate through western processes. From this te ao Māori perspective, becoming literate is deeply rooted in intergenerational input and knowledge sharing; it is reliant on both experiencing the world as an individual and as a member of groups. It encompasses both the physical and abstract, and at its heart is a deep desire to continue building knowledge about the complex workings of the universe and our role as humans across generations in that process.

This assertion essentially defines what it is to mātau. The ability to weave philosophical and epistemological understandings into useful, effective and informed everyday practice. Therefore, mātauranga (literally, mātau is to know, ranga is to weave) is the capacity to internalise understanding, making it a part of yourself through understanding and use. Mātau/mātauranga is committing true/deep/thorough understanding to the sub-conscious in order to have the capacity to attend to learning new understandings and knowledge.

## Ka Ora: Flourishing

Ka Ora is to be well, to thrive, to flourish, to be and become oneself. When students feel well and have been given opportunities to express their mana tangata, they are more open to learning from and about knowledge systems beyond their own.

As discussed throughout this paper, the notion of a connected, well child, is reliant on a vast range of elements. Being aware, knowledgeable, and secure in the world makes the Ka Ora phase possible.

There are some hapū that have children of all ages...put in a cluster and started teaching. Those who are bright, whether they were male or female, were taken out and you started another form of teaching. Those who were found to have super bright intellect were taken out and taught on their own and that became the exclusive wānanga system. (Sir Patu Hohepa)

The traditional Māori pedagogy described by Sir Patu Hohepa is an illustration of the intensity of teaching practices from a te ao Māori perspective. In this example, learning experiences were a social and intergenerational undertaking, but at the same time, very close attention was paid to each individual child. This close attention and observation of the individual is a pedagogical practice referred to in the narratives we have relied on in this paper. The level of observational skill involved is an art that is arguably incomprehensible to modern minds that are so reliant on external modes of recording. According to this approach knowing the learner involves understanding personal disposition, the analysis of characteristics displayed by children, the interactions between child and others, and the interactions between the child, their environment, and their whakapapa.

By widening the view of literacy, we assert that literacies, as processes of tiritiria, require high-level capabilities and expertise. By arguing that we need to engage with indigenous ways of knowing, and accessing knowledges passed on through oral, written and visual texts we have sought to reframe the status quo view of literacy. Teaching of literacies is broader than imparting knowledge of encoding and decoding print text. It requires utilising child’s current language capabilities to build more complex textual and knowledge expertise, not only in finding the learnings in languages and texts but also knowing how these are employed in socio-cultural contexts. From this perspective, children are empowered to use their wide arrays of cultural and linguistic resources as tools for learning and participating.

## He whakaaro whakakapi—Concluding thoughts

Tiritiria, a Māori philosophical belief about the nature of knowledge, has been used to push back on the heavy reliance of New Zealand education on western concepts and definitions of literacy, and to frame our argument for the re-conceptualisation of literacies in Aotearoa New Zealand. According to our understanding of tiritiria, re-conceptualising literacies also requires re-positioning children as maurea and reclaiming traditional Māori bodies of knowledge to inform current literacy definitions and practices. Perceiving children as maurea means seeing them as whole, which therefore requires consideration of an individual’s generational connections to past, present, and future, and an acknowledgment that all children possess inherent and inherit-ed literacies.

We have utilised in ancient whakataukī as a context from which to discuss and illustrate our developing conceptualisation of literacies. We argue that this conceptualisation has the capacity to reframe both literacies and play in Aotearoa New Zealand. Contributions from Te Tai Tokerau communities, analysed using the Mana Model (Webber & Macfarlane, 2020), has enabled place-based knowledge and expertise from a Māori perspective to support the emergence of this theoretical frame. This framework necessarily critiques and questions the presence of Eurocentric views of literacies prevalent in the New Zealand school system. Considering historical injustices and current systemic challenges in education, the framework allows for an expansion of ideas around literacies, literacy experiences and play.

The whakataukī that provides the structure for our framework has transformational potential. By empowering connectedness and synergy within and between the phases of the framework, as well as within and between people, places, and knowledge bases. Although sequenced for the purpose of this paper, the phases are interconnected and dynamic. They do not always reflect a linear process or progression in a certain direction, rather, there is a process of moving in and out of appropriate phases based on the child and their strengths. This function of the framework also reflects the belief presented through tiritiria that literacies are multitudinous, reiterating the multiplicity of what can be learned, and the ways knowledge can be shared and generated. Such movement and engagement with the framework supports the empowerment of children to ora (flourish) by realising their potential through exploration, connection, relationships, awareness, application and incorporation.

Maurea, the most treasured resource of all iwi are capable of traversing diverse settings, have the ability to communicate clearly in any mode they choose, be intuitive, be respectful and be respected. They know who they bring with them, and who they are responsible to. Maurea will have a positive impact on their worlds. We maintain that engaging in empowering indigenous literacies will enable maurea to become exactly who they are—themselves.
